# Plasmonic mode coupling and thin film sensing in metal–insulator–metal structures

**DOI:** 10.1038/s41598-021-94143-2

**Published:** 2021-07-23

**Authors:** N. Andam, S. Refki, S. Hayashi, Z. Sekkat

**Affiliations:** 1 Department of Chemistry, Faculty of Sciences, University Mohammed V, Rabat, Morocco; 2 Optics and Photonics Center, Moroccan Foundation for Advanced Science and Innovation and Research, University Mohammed VI Polytechnic, Rabat, Morocco; 3 Graduate School of Engineering, Kobe University, Kobe, 657–8501 Japan; 4 Department of Applied Physics, Osaka University, 2-1 Yamadaoka, Suita, Osaka 565-0871 Japan

**Keywords:** Engineering, Materials science, Nanoscience and technology, Optics and photonics, Physics

## Abstract

Optical sensors based on surface plasmon resonance (SPR) in the attenuated total reflection (ATR) configuration in layered media have attracted considerable attention over the past decades owing to their ability of label free sensing in biomolecular interaction analysis, and highly sensitive detection of changes in refractive index and thickness, i.e. the optical thickness, of thin film adsorbates (thin film sensing). Furthermore, SPR is highly sensitive to the refractive index of the medium adjacent to the bare metal, and it allows for bulk sensing as well. When deposited at the metal/air interface, an adsorbed layer disturbs the highly localized, i.e. bound, wave at this interface and changes the plasmon resonance to allow for sensing in angular or wavelength interrogation and intensity measurement modes. A high degree of sensitivity is required for precise and efficient sensing, especially for biomolecular interaction analysis for early stage diagnostics; and besides conventional SPR (CSPR), several other configurations have been developed in recent years targeting sensitivity, including long-range SPR (LRSPR) and waveguide-coupled SPR (WGSPR) observed in MIM structures, referred here to by MIM modes, resulting from the coupling of SPRs at I/M interfaces, and Fano-type resonances occurring from broad and sharp modes coupling in layered structures. In our previous research, we demonstrated that MIM is better than CSPR for bulk sensing, and in this paper, we show that CSPR is better than MIM for thin film sensing for thicknesses of the sensing layer (SL) larger than 10 nm. We discuss and compare the sensitivity of CSPR and MIM for thin film sensing by using both experiments and theoretical calculations based on rigorous electromagnetic (EM) theory. We discuss in detail MIM modes coupling and anti-crossing, and we show that when a thin film adsorbate, i.e. a SL), is deposited on top of the outermost-layer of an optimized MIM structure, it modifies the characteristics of the coupled modes of the structure, and it reduces the electric field, both inside the SL and at the SL/air interface, and as a result, it decreases the sensitivity of the MIM versus the CSPR sensor. Our work is of critical importance to plasmonic mode coupling using MIM configurations, as well as to optical bio- and chemical-sensing.

## Introduction

Surface Plasmon polaritons (SPPs) and localized surface plasmons (LSPs) have been reported for more than a century ago^1,2^, and they constitute the ground base work of current research in the field of plasmonics, whereby the electric field of light sets free electrons of metals into oscillatory type motion, at optical frequencies, and allows for a strong field enhancement (FE) and highly localized, i.e. bound, waves at surfaces of metals and interfaces of metals and dielectrics^3,4^. The field enhancement and bound character of surface plasmons (SPs) paved the way, among other applications, to the development of plasmonic sensors^5–7^. Indeed, SPs are highly sensitive to any change of the metal/dielectric interface, i.e. boundary, such as the adsorption of molecules to the conducting surface^8,9^, thereby allowing for thin film sensing and characterization^10^, and SPs waves penetrate to different extents, i.e. penetration depths, into metals and dielectrics, allowing for sensing of changes in the refractive index of media thicker than the SPs penetration depths^11–15^. In this regard, LSPs at metal nanoparticles are able to perform sensing for volumes 40–50 times smaller than SPs sensors using thin metal films, and thereby are much less sensitive to bulk refractive index changes. The plasmon decay length, $\delta$, in the sensing medium, are much shorter for nanoparticles versus thin films. In fact, $\delta$ for nanoparticle are in the 5–10 nm range, while for SPs at metal films it is larger than 100 nm^16^. The sensitivities of LSPs and SPs are comparable in the short-range regime, i.e. in a range not exceeding the decay length of plasmons  at the nanoparticle / sensing medium interface^17^. Long-range SPs^14^ and MIM^12^ modes are most sensitive to bulk sensing owing to large penetration depths, i.e. in the micrometer range. Plasmonic sensors found extensive use in the areas of food inspection^5,18,19^, medical diagnostics^20,21,22^, drug screening^23,24^, and observing and controlling photoswitching phenomena by refractive index change $(\Delta n)$^25–27^.

Various plasmonic sensors based on multilayer structures, in the attenuated total reflection (ATR) configuration, have been developed due to the ease of fabrication, and capability of real time investigation of molecular interaction, for example, biomolecular interaction analysis. In this paper, we develop and study two types of SPR sensors based on ATR in the Kretschmann geometry for thin film sensing, and given that the main application of the sensors is bio-sensing, i.e. biomolecular interaction analysis, and to focus on the sensitivity of the sensors discussed, this paper assesses the sensitivity of the developed sensors, by using a photochemical method to modulate the refractive index of the sensing medium artificially. We use an azobenzene derivative, i.e. disperse red one (DR1), which is well known to undergo switching via reversible trans–cis isomerization upon absorption of visible light^28,29^. When such a dye is embedded into a material, for example, a polymer, it offers the possibility of switching the refractive index of the polymer by the dye’s isomerization. In fact, azo-dye containing materials, i.e. for example, DR1-doped poly-methyl-methacrylate (DR1/PMMA) have been studied over the past decades for possible applications in all optical switching and data storage and holographic recording^30–32^, as well as photomechanic transduction^33–38^. In fact, thin films of DR1/PMMA act as transducers that can convert incident light into a change of molecular polarizability and $\Delta n$, and in our previous studies, we used this property of the azo dyes for tuning Fano resonances in a multilayer stack constituted by a metal and two dielectrics by light absorption of DR1 molecules imbedded into a PMMA host. The DR1/PMMA layer was positioned as the outermost layer of our Fano structure^25,39^, and other researchers studied the performance of a long range layered plasmonic structure containing DR1/PMMA as the outermost layer which functioned as an all-optical switch^40^.

In this paper, we use DR1/PMMA as a thin film adsorbate on a metal surface to study thin film sensing. We use two plasmonic structures: metal–insulator–metal (MIM) and conventional SP resonance (CSPR), and we show that CSPR sensing is superior to that of MIM for thin film sensing for thicknesses of the SL larger than 10 nm. Sensing is performed by observing the shift of the MIM and the CSPR modes via, photochemically induced ${\Delta n}_{SL}$ of the sensing layer (SL). Our approach is to exploit the potential of the photofunctional DR1 molecules, embedded in the SL, in realizing active photochemical modulation of the resonances of both plasmonic systems, using blue light irradiation to excite the DR1 molecules and induce photoisomerization. Based on both theoretical and experimental results, we demonstrate that the deposition of a thin dielectric film, i.e. SL, on the top of the outermost metal of the MIM structure modifies the coupled modes of this structure, thereby, reducing significantly the optical electric field inside the SL as well as at the SL/air interface, and, as a result, decreasing its sensitivity. While in the previous studies^12,41,42^, MIM sensing was shown to be $\sim 7.5$ more sensitive than CSPR for bulk sensing, the present study shows that CSPR is better ($\sim 3-5$ times better depending on the thickness of the sensing layer (SL) than MIM for thin film sensing (vide infra).

We will first discuss theoretically, i.e. by calculating the electric field distribution inside the SL and at the SL/air interface, the effect of the thickness of the sensing layer (SL) on field enhancement in both MIM and CSPR structures; and we conclude that, for both structures, the thicker the SL the smaller the enhancement. We also estimate the sensitivity, depending on the thickness of the SL, given a ${\Delta n}_{SL}$ induced by external stimulus, which is light irradiation here. To confirm experimentally the theoretical predictions, we prepared MIM as well as CSPR chip sensors and we coated them with a sensing layer having the smallest possible thickness, imposed by our experimental conditions, to obtain the largest, experimentally observable, field enhancement and sensitivity. ${\Delta n}_{SL}$ was photochemically induced in both MIM and CSPR structures, and it was controlled by the energy dose absorbed by the SL upon light irradiation and photoisomerization of the DR1 molecules. Thereby we could observe mode shifts in both structures and compare their sensitivities; we found experimentally that CSPR is more sensitive than MIM. We also studied the real time photo-induced change of ${\Delta n}_{SL}$ depending on the polarization of the irradiation light and found that they are strongly polarization dependent, owing to photo-selection and photo-orientation of the DR1 molecules, in agreement with a long literature^ 25,43,44,45^.

## Theoretical calculations

### Electromagnetic calculations of Field Enhancement of MIM and CSPR sensors

**Figure 1:**
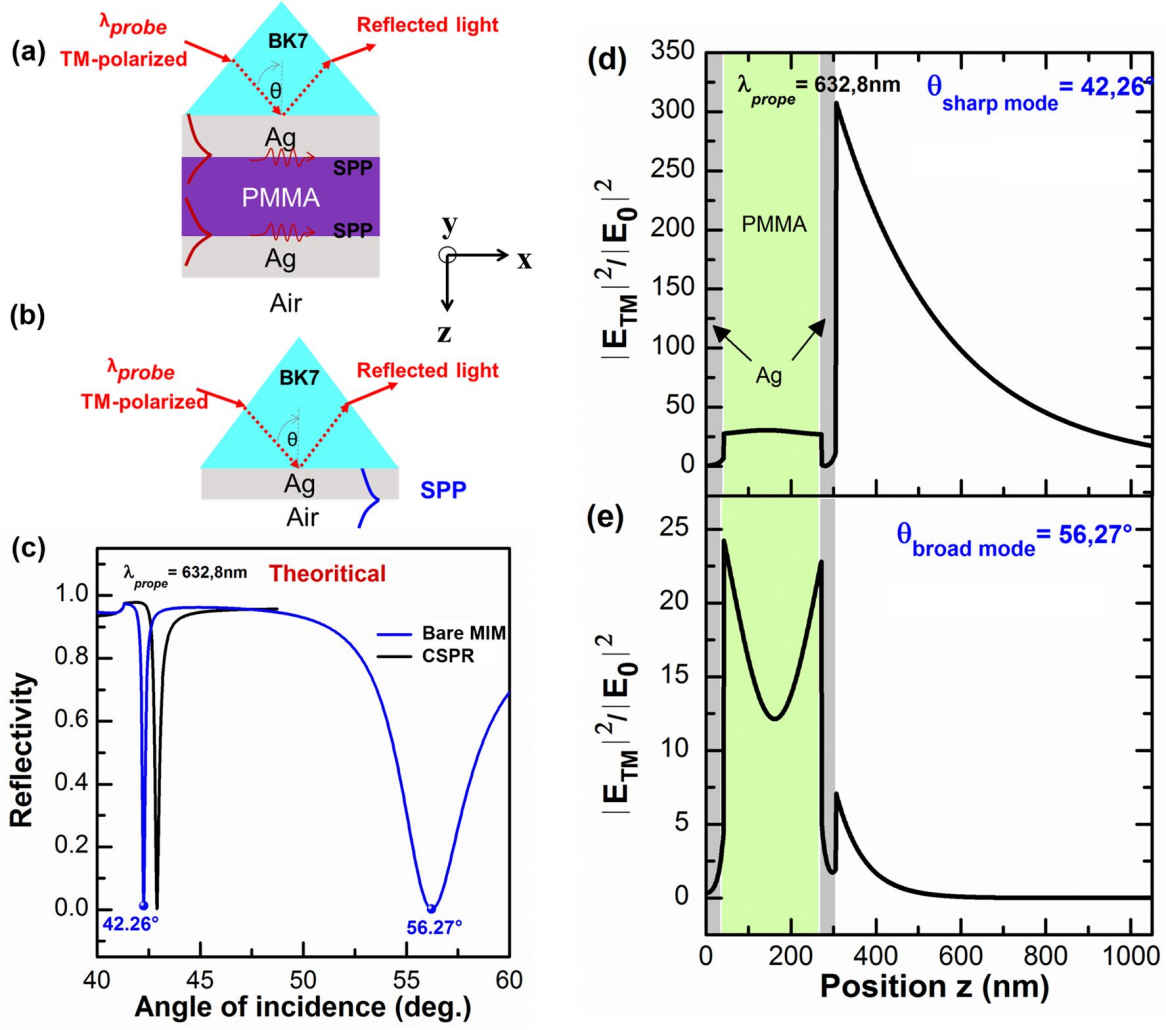
Calculations of $\theta$-scan ATR and electric field profiles. Schematic configuration of (**a**) the MIM stack consisting of PMMA sandwiched between 2 Ag layers, and (**b**) the CSPR sensor based on silver. The outer medium in both structures is air. and *x*, *y*, and *z* correspond to the system coordinates (**c**) $\theta$-scan ATR calculations of MIM and CSPR structures without the sensing layer (SL); and (**d**, **e**) Electric field profiles of the MIM structure calculated at the sharp and broad modes resonance angles, i.e. at 42.26° and 56.27°; respectively.

The MIM and CSPR sensors are schematically depicted in Fig. 1. Figure 1a shows the MIM structure consisting of a coupling prism, as the first layer, and a PMMA insulator layer sandwiched between two Ag metal layers; and the CSPR sensor is shown in Fig. 1b, and it is composed of the coupling prism layer and a Ag layer. The medium on top of the outermost layer of both structures is air. We performed electromagnetic (EM) calculations of electric field (EF) profile, together with the $\theta$-scan ATR calculations based on Fresnel reflection and transmission coefficients, with and without the DR1/PMMA SL with a thickness, $d$, varying from $0$ (bare metal surface) to $70 \, {\text{nm}}$. These calculations allow for the determination of theoretical spectra of multi-layer structures corresponding to MIM and CSPR by varying the structural parameters, i.e. complex refractive indices and thicknesses of the different layers composing the structures. For the BK7 prism, we used ${n}_{BK7}=1.5151$ at a wavelength of ${\lambda }_{probe}=632.8 \, {\text{nm}}$, from a database^46^, and we searched for refractive indices and thicknesses of Ag, PMMA, and DR1/PMMA that lead to the optimum sensor performance. The respective refractive indices values we found for Ag and PMMA and DR1/PMMA are ${n}_{Ag}=0.0719+i4.1430$, ${n}_{PMMA}=1.4889+i0.0094$, ${n}_{DR1/PMMA}=1.5650+i0.012$; respectively, and the thicknesses of the different layers of the bare MIM structure are $41$, $230$, and $34 \, {\text{nm}}$, for the first Ag, PMMA, and second Ag layers, respectively; and the thickness of Ag used for the ideal CSPR structure is $52 \, {\text{nm}}$. We used a 12% weight concentration of DR1 with respect to PMMA, and the complex refractive indices of the different materials used in our structures are identical to those reported in 10 (vide infra). The results of the calculations are shown in Fig. 1c–e.

Figure 1c is the $\theta$-scan of the ATR spectra obtained for the MIM and CSPR sensors without the SL. Note that the MIM structure exhibits a sharp ATR mode around 42.26° and a broad mode around 56.27°. The purpose of this section of the study is to discuss the nature of the modes of the MIM structure and to show that the sharp mode is appropriate for thin film sensing, and compare the characteristics of the sharp mode to those of the CSPR mode. Indeed, the sharp mode is located at an angle lower than that of CSPR, and the full width at half maximum (FWHM) of the CSPR mode is two times larger than that of the MIM sharp mode. According to our previous study^12,47^, a MIM structure with a semi-infinite metal bears both symmetric (S-SPP), and antisymmetric (AS-SPP) modes that arise from the coupling of the two SPP modes localized at MI interfaces of the MIM structure (Fig. 1a), assuming that the insulator layer is thin enough. When the metal layers have finite thicknesses, the MIM structure sustains also an SPP mode localized at the outer Ag/Air, interface^12,47^. S-SPP and SPP couple to each other through their evanescent fields inside the Ag layer, and lead to hybrid modes; i.e. a sharp and a broad modes. We performed EM calculations of the EF distributions of our MIM structures by the 2 × 2 transfer matrix method^48^. The square of the amplitude of the electric field normalized to that of the incident light $\left({\left|{E}_{TM}\right|}^{2}/{\left|{E}_{0}\right|}^{2}\right)$ is plotted as a function of the position $z$ of the bare MIM structure (Fig. 1d,e) at the incident angles 42.26° and 56.27° corresponding to the sharp and the broad resonances dips; respectively (Fig. 1c). Figure 1d,e as well as electric field profiles shown in Supporting Information, show that the MIM modes are hybrid in nature indeed. The electric field of the sharp dip is highly enhanced at the Ag/air interface and decays exponentially in air away from the interface. Judging from the electric field profile, the sharp mode is basically a SPP mode localized at the Ag/air interface, referred hereto by Ag/Air-SPP mode. The electric field profile of the broad dip (Fig. 1e) consists of an amplitude inside the PMMA layer larger than the weak exponential tail in air, and the profile inside the PMMA layer is typical of the S-SPP. The light energy inside and outside the PMMA layer is weaker for the broad dip versus the sharp dip. In particular, the field enhancement is ~ 60 times larger at the Ag/air interface for the sharp versus the broad dip, and consequently, the sharp resonance mode, with its substantially enhanced evanescent wave in air, can be used for sensing applications^47,49^. While the suitability of this mode was proven for bulk sensing^12,13^, this paper aims to assess its capabilities for thin film sensing , and therefore only the sharp mode of the MIM structure is considered for the comparison with the well-established CSPR thin film sensing^27,50^.

**Figure 2.**
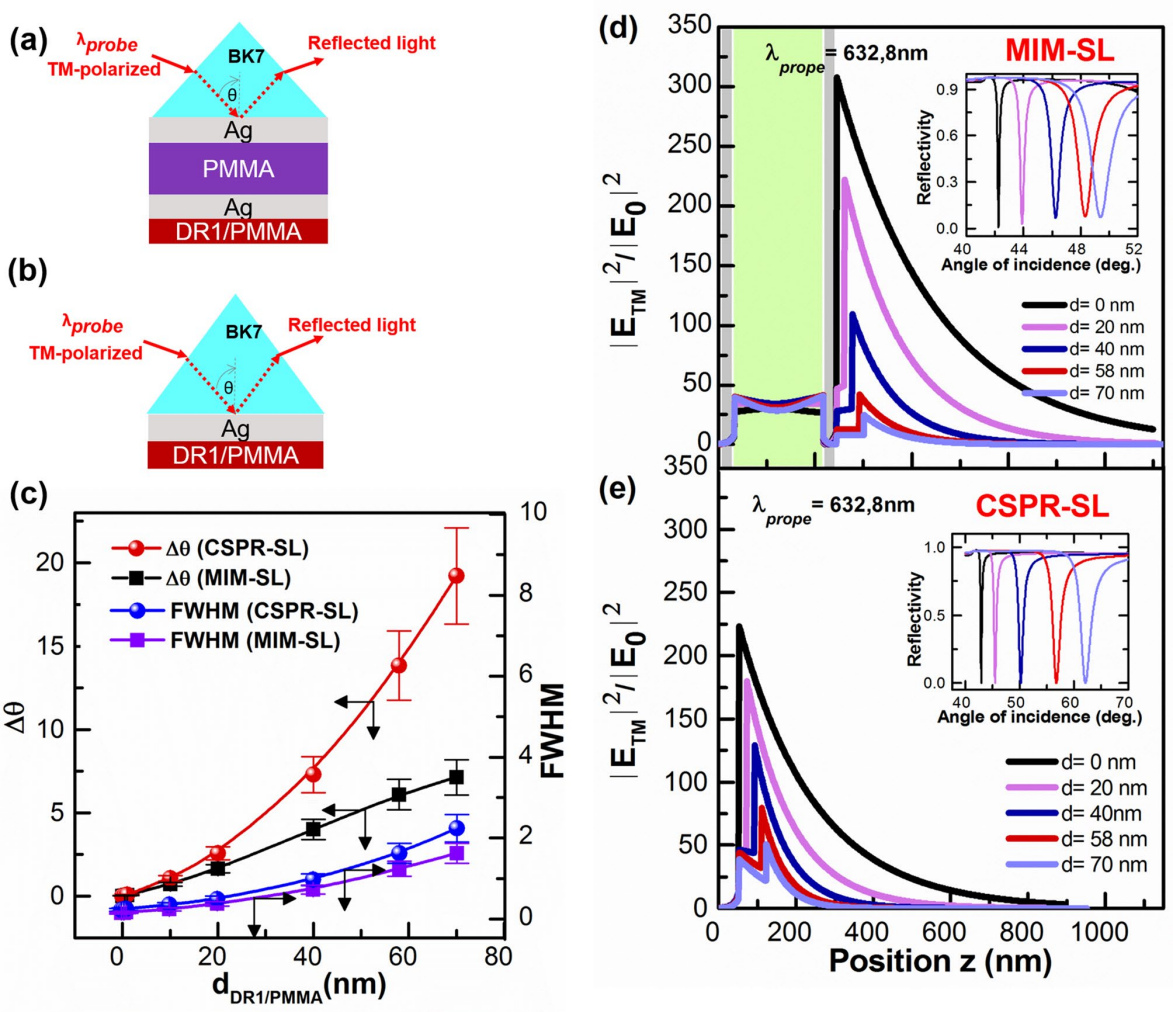
(**a**, **b**) schematic of the MIM and CSPR structures, (**c**) mode shift and FWHM dependence on the thickness of the sensing layer (SL) for both structures; (**d, e**) Electric field profiles calculations for different thicknesses of the sensing layer (SL) at (**d**) the sharp MIM mode and (**e**) the CSPR mode. The insets of (**d**, **e**) are ATR $\theta$-scan calculations.

For thin film sensing, an adsorbate layer, i.e. the SL, is deposited on top of the structure; a feature which disturbs the field distribution (FD) and changes field enhancement (FE) at the structure and, consequently, they need to be recalculated. Figure 2 shows the results of EM calculations of the field distribution and field enhancement for both the MIM and CSPR structures when a sensing layer is deposited on top of the structure*.* The inset to Fig. 2d and e show $\theta$-scan ATR spectra of the sensor (MIM for (d) and CSPR for (e)) for different thicknesses of the SL, and the thicker the SL, the larger the mode shift. These figures also show that the FE at the Ag/air interface is larger for MIM versus CSPR without the SL $(d=0)$, i.e. by a factor of $\sim \mathrm{1,4}$. The field enhancement of MIM starts diminishing compared to that of CSPR for thicknesses of the SL around $40 \, {\text{nm}}$. Furthermore, the comparison of the resonance dip shift $(\Delta \theta )$ of the two structures (Fig. 2c) demonstrates that $\Delta \theta$ of CSPR is higher than that of MIM for thicknesses exceeding $10 \, {\text{nm}}$. Figure 2c also shows the evolution of the FWHM of the sharp MIM and CSPR modes as the thickness of the SL increases. FWHM of both structures is comparable for the same thickness of the sensing layer (SL), with a noticeable, i.e. small, difference at larger thicknesses.

According to our detailed studies (summarized in Supporting Information), in the absence of coupling, i.e. for an uncoupled MIM structure having a semi-infinite outer metal layer, the S-SPP mode stays at the same position ($52.81^\circ$), while the Ag/Air (or Ag/SL/Air) mode shifts from $42.78^\circ$ to $62.51^\circ$ as the SL thickness increases from 0 to 70 nm, as demonstrated for the CSPR structure. As the SL thickness increases, the detuning of the modes (difference in the resonance positions) decreases first, becomes zero around $d=48 \, {\text{nm}}$ and turns to increase as the SL thickness increases further. In the MIM structure considered above, the coupling of the S-SPP mode with the Ag/SL/Air mode results in hybridized modes constructed by in-phase (symmetric) and out-of-phase (antisymmetric) superpositions of respective modes. Around the crossing point (zero detunings at $d=48 \, {\text{nm}}$), the modes are pushed away from each other because of the repelling of the modes (see Fig. S1 of Supporting Information) (anticrossing behavior)^47,51^. The shift of the sharp dip toward a lower angle seen in Fig. 1(c) is indeed due to the repelling of the mode. The mode shift, i.e. $\Delta \theta$, strongly depends on the angular position of the resonance^52^, that is the reason why $\Delta \theta$ are different for CSPR and MIM. As can be seen in Figs. S2 and S3 in Supporting Information, relative contributions of the S-SPP and Ag/SL/Air modes to the coupled modes depend strongly on the detuning of the mode, especially around the crossing point. Drastic changes in the EF profile of the MIM structure, with the change of the sensing layer (SL) thickness, seen in Fig. 2d are understood as the changes in the relative contributions of the S-SPP and Ag/SL/Air modes brought by the change in the detuning. The sensitivity of both sensors is discussed next.

### Sensitivity calculations of the MIM and CSPR sensors

**Figure 3:**
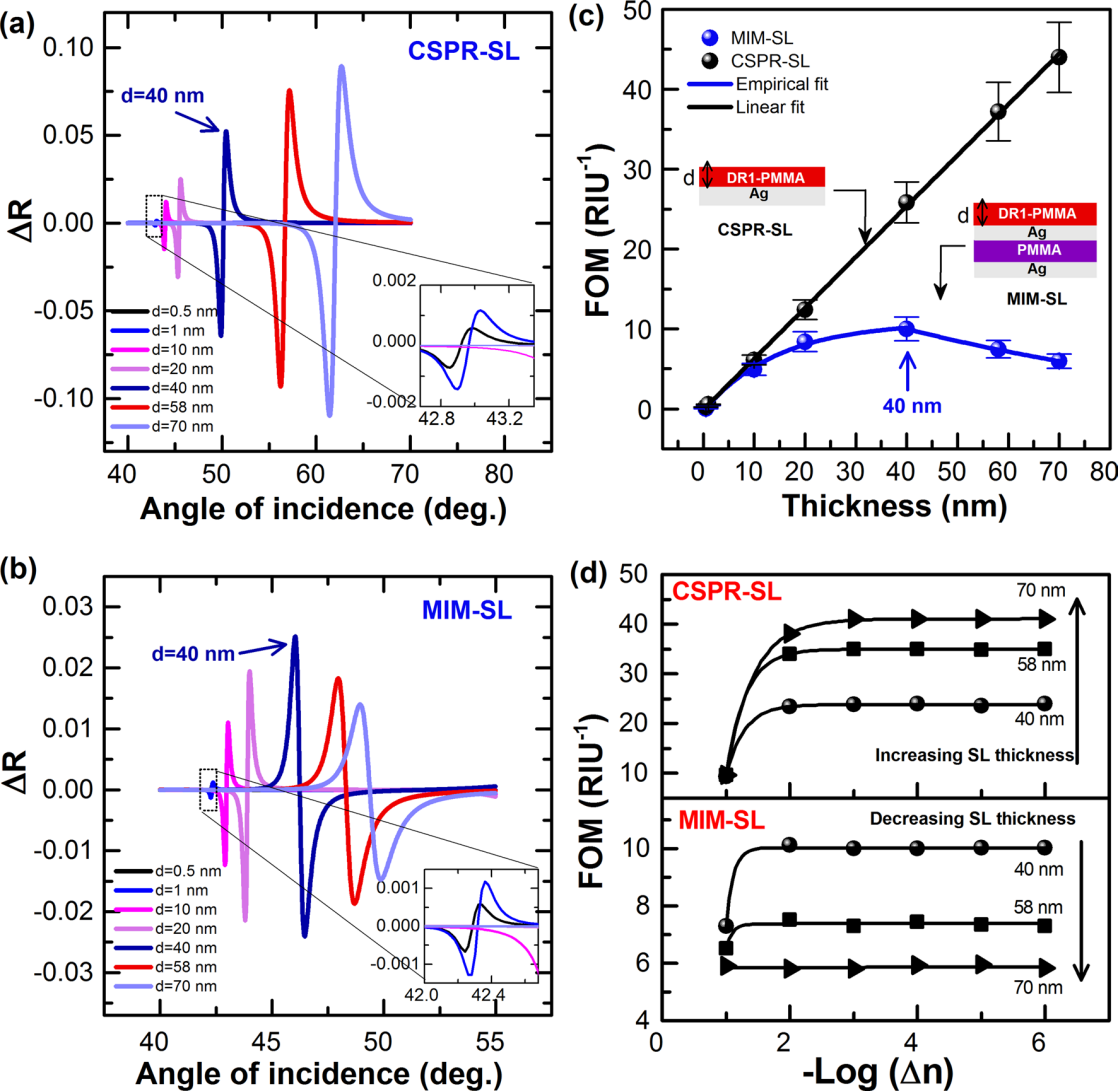
$\Delta R$ as a function of $\theta$-scan of (**a**) CSPR and (**b**) MIM sensors. The insets represent the zoom-in of $\Delta R$ calculations for very small thicknesses, e.g. $0.5 \, {\text{nm}}$ and 1 nm for both sensors. FOM calculations of MIM and CSPR sensors depending on (**c**) the thickness d of the sensing layer (SL) and (**d**) the refractive index change for three different thicknesses of the sensing layer (SL) as indicated in the figure. In (**c**) the arrow at 40 nm points to the SL thickness for the optimized MIM-SL structure, and in (**d**) the arrows point to the direction of change of the SL thickness.

To calculate the sensitivity of the CSPR and MIM sensors, we first use $\Delta n\sim 2.5\times {10}^{-3}$ for the DR1/PMMA sensing layer obtained from the photoswitching experiments at the steady-state of pump light for a typical irradiation dose (vide infra), and we calculate the corresponding change in the reflectivity $\Delta R$ as a function of, $\theta$, as shown in Fig. 3a,b, for both CSPR (Fig. 3a) and MIM (Fig. 3b) sensors. $\Delta R$ is defined as the difference between the curves in the dark and at the steady state of irradiation (Eq. 1), and it is calculated for different thicknesses of the SL as indicated in Fig. 3a,b.

1 $$\Delta R={R}_{ON}-{R}_{OFF}$$

For the CSPR sensor, $\Delta R$ increases with the increasing thickness of the SL, within the thickness range considered, and for the MIM sensor, it increases and reverses at ${d}_{SL}=40 \, {\text{nm}}$. Given that the position of the resonance dip shifts more in the case of CSPR than for MIM and that the FWHM is comparable, for each thickness, for both structures (Fig. 2c), a figure of merit (FOM) of the sensors needs to be calculated as a function of the thickness of the sensing layer (SL). FOM depends on the position of the resonance angle and the FWHM of the mode, and it characterizes the maximum achievable sensitivity, by intensity modulation (${S}_{I}$) for example, and it is given by^53,54^

2 $$FOM={max}_{{\theta }_{res}}(\Delta R\left({\theta }_{res}\right)/\Delta n)$$

with ${S}_{I}=(\Delta R\left({\theta }_{res}\right)/\Delta n)$. In our ATR calculations, $R(\theta )$ corresponds to the reflectivity as a function of the angle of incidence at the resonance condition ${\theta }_{res}$, and $\Delta R$ is given by Eq. (1). The calculated FOM of both sensors, i.e. CSPR and MIM (sharp mode), using Eqs. (1–2) for different thicknesses of the sensing layer (SL) is shown in Fig. 3c. This figure shows that FOM of both sensors is the same for small thicknesses of the SL, i.e. smaller than or equal to $10 \, {\text{nm}}$, and they depart from each other as the thickness of the SL layer increases further than $10 \, {\text{ nm}}$, with better sensitivity for the CSPR versus the MIM sensor. A blow up of the data in the 0–10 nm range (not shown) shows that the FOMs for CSPR and MIM are nearly the same. We also calculated FOM for both sensors as a function of ${\Delta }_{{n}_{SL}}$ for 3 different thicknesses as shown in Fig. 3d, and we found the same conclusion as above. That is increasing the thickness of the SL decreases the sensitivity of MIM and increases that of CSPR; for ${\Delta }_{{n}_{SL}}$ absolute values larger than 0.1, FOM is constant for a given thickness. From the above analysis, we conclude that, in theory, CSPR is better than MIM for thin film sensing for thicknesses of the SL exceeding $10 \, {\text{nm}}$. This is what is confirmed experimentally next.

## Materials and methods

### MIM and CSPR sensors preparation

The SL-MIM stack which we fabricated, according to the theoretically optimized structure, and studied, is schematically depicted in Fig. 2a; it was prepared as follows. First, a film of Ag was deposited on a BK7 cleaned glass substrate by vacuum evaporation, then on top of this film, an insulator layer of PMMA was spin-coated from a chloroform solution, with 2 wt% concentration ($0.30 \, {\text{g}}$ of PMMA dissolved in $10 \, {\text{ml}}$ of chloroform) with a speed of $3300 \, {\text{rpm}}$ for $60 \, {\text{s}}$, and the prepared stack of Ag/PMMA was heated on a hot plate at $80^\circ {\text{C}}$ for $45 \, {\text{min}}$. Finally, another film of Ag was deposited on top of PMMA to complete the MIM structure. The thicknesses of the PMMA and Ag layers are close to the theoretically optimized MIM structures, and the metal film thicknesses were monitored by a quartz microbalance (QMB). In addition to the MIM structure, we prepared a CSPR structure with a single layer of Ag vacuum evaporated on a BK7 cleaned glass substrate. For the sensing layer (SL), $58 \, {\text{nm}}$ films of DR1/PMMA were spin-coated on top of both MIM and CSPR structures. For the preparation of sensing layer, DR1, commercially available, was mixed with the PMMA chloroform solution with a concentration of 12 wt% of DR1 relative to PMMA, and films of DR1/ PMMA were spin-coated on top of the structures with a rotation speed of $5500$ for $60 \, {\text{s}}$. The films were dried at $80^\circ {\text{C}}$ for $45 \, {\text{min}}$ to remove the remaining solvent. The film thicknesses deposited by spin-coating were measured by a surface profilometer (Bruker). For UV–Vis absorption measurements, the films were spin-coated directly on top of cleaned glass substrates, in the same conditions, i.e. the same thicknesses for PMMA and DR1/PMMA, and the absorbances were measured using a UV–Vis spectrophotometer Perkin Elmer-Lambda 1050. The optical density of the DR1/PMMA film thus prepared was ${OD}_{488nm}\sim 0.08$ at the maximum absorption wavelength (spectrum not shown).

### Optical set-up of ATR measurements and in-situ photochemistry

To measure the angle-scan ATR spectra in a Kretschmann configuration for both MIM and CSPR structures, we used a custom made optical setup. The prepared MIM and CSPR structures were put, separately, on the bottom surface of a $90^\circ$-BK7-prism, using an index matching oil, and mounted on a ($\theta$–2$\theta$) rotating stage. The prism-sample system was illuminated by transverse magnetic (TM or P) polarized light from a Helium–Neon (He–Ne) laser operating at a wavelength of ${\uplambda }_{\mathrm{probe}}=632.8 \, \mathrm{nm}$ as a probe beam. The power of the probe beam was kept to a few µW to avoid photoreaction in the sample, and its size was $\sim 1 \, {\text{mm}}$ at the surface of SL. The light reflected at the base of the prism was measured, as a function of the angle of incidence, by using a Si photo-diode connected to a lock-in-amplifier. The precision of the measurements of the incidence angle was less than $2^\circ \%$, i.e. $0.018^\circ ;$ a feature which causes an error in reflectivity measurements of $\sim 7\times {10}^{-3}$, and the precision on the measurements of the real and imaginary parts of the complex refractive index by our SPR machine is $4\times {10}^{-4}$. Details on the experimental set up can be found in 10. To perform in-situ photoisomerization experiments during reflectivity scans, we used an irradiation laser beam, operating at a wavelength of ${\uplambda }_{\mathrm{pump}}=488 \, \mathrm{nm}$, from a $50 \, {\text{mW}}$ frequency doubled, diode-pumped semiconductor laser. The power at the surface of the SL could be adjusted with neutral density filters. The pump beam was $\sim 6 \, {\text{mm}}$ in diameter and it was centered on the probe beam and irradiated the plasmonic structure from the back perpendicularly to the plane of the structure. The pump wavelength is close to the absorption maximum of the SL and efficiently induces photoisomerization of DR1^10,27,28^.

## Results

### Effect of an adsorbate layer on the sensitivity of the MIM and CSPR structures

The theoretical calculations of the previous section show that when an adsorbate thicker than $10 \, {\text{nm}}$ is added on the top of the structure, the CSPR mode is shifted more than the MIM mode, and as a result, adding an adsorbate layer on the top of the plasmonic structure decreases the sensitivity of MIM versus CSPR. To confirm these results, we deposit a sensing layer (SL) on top of the plasmonic structure, with a thickness $d=58\pm 4 \, {\text{nm}}$, which is close to the theoretical thickness that maximizes the FOM of the MIM sensor, i.e. $40 \, {\text{nm}}$ (Fig. 3c). This is the minimum thickness that can be obtained given our experimental conditions, i.e. a thickness below which the surface of the sensing layer (SL) deposited by spin-coating is non-homogenous.

**Figure 4:**
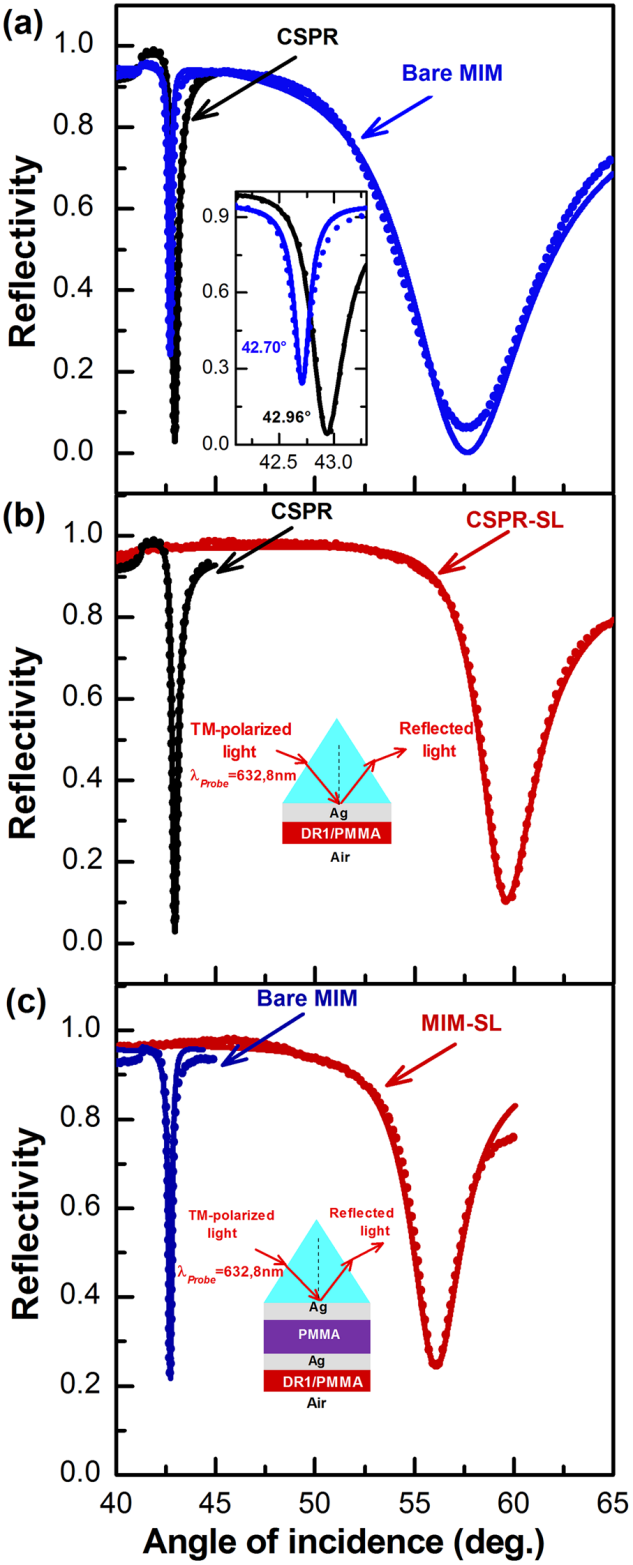
Angle-scan ATR spectra in a Kretschmann configuration for CSPR and MIM. (**a**) Both CSPR and MIM, sharp and broad, modes without the SL. (**b**) CSPR and (**c**) MIM, with and without the DR1/PMMA sensing layer. The dots are experimental data and the solid lines are theoretical Fresnel fits. The inset in (**a**) is an expanded view of the resonance region of the CSPR and Sharp MIM mode, and in (**b**, **c**) are the schematics of the CSPR and MIM structures; respectively.

Figure 4 shows the TM-ATR spectra as a function of the incidence angle which we obtained for the samples of MIM and CSPR structures, prepared without and with the $58 \, {\text{nm}}$ adsorbate. Figure 4a shows that the ATR spectrum of the bare MIM structure, exhibits a sharp resonance dip at a lower incidence angle compared to the mode of bare CSPR structure, and the FWHM for MIM is smaller than that of CSPR, i.e. $\sim 0.22^\circ$ for MIM versus $0.34^\circ$ for CSPR. Figure 4b,c show the $\theta$-scan ATR spectra measured for CSPR and MIM sharp mode without and with the DR1/PMMA adsorbate film; respectively.

The resonance dip of the bare CSPR sensor is located at $42.96^\circ$; and after adding the adsorbate layer of $58 \, {\text{nm}}$ of DR1/PMMA, the resonance shifts toward a higher incidence angle, i.e. $59.67^\circ$ (Fig. 4b). Similarly, for the MIM structure, the $58 \, {\text{nm}}$ adsorbate layer shifts the resonance to higher angles, i.e. from $42.70^\circ$ to $56.03^\circ$ (Fig. 4c). Both MIM modes shift to higher angles, and only the low-angle mode is shown. The high-angle mode is shifted out of range for our setup and cannot be recorded. The experimental results of the ATR spectra of both MIM and CSPR are in good agreement with the theoretical Fresnel fits. The complex refractive index of the inner Ag layer, as well as the complex refractive index and thickness values of the adsorbate layer, i.e. DR1/PMMA, extracted from the fitted data, are the same for both structures, and Table 1 shows the data relative to MIM only. This table also shows that the thicknesses of the different layers extracted from the theoretical Fresnel fits of the light reflectivity at the multilayer structures, i.e. experimental thicknesses, are slightly different from the values corresponding to the theoretically optimized structure. We found that during the fitting procedure of the MIM-SL structure, a 1 nm change in the thickness of the PMMA sandwich layer and the outer Ag layer lead to $\sim 0.12^\circ$ and ~$0.25^\circ$ increase in the minimum position of the sharp MIM mode, respectively. The thickness of PMMA and that of the outer Ag layer, including its complex refractive index, have a strong influence on mode coupling and the resonance positions of the low- and high-angle modes. Next, we experimentally impose the same refractive index change of the SL, i.e. the adsorbate, by photoisomerization of DR1, in both MIM and CSPR structures, and we estimate their sensitivities for mutual comparison.

**Table 1:** Complex refractive index, at $632.8 \, {\text{nm}}$, and thickness of the inner and outer layers of Ag, the PMMA sandwich layer, and the DR1/PMMA sensing layer.

Structure	Theoretical			Experimental data					
	MIM-SL*			Bare MIM			MIM-SL		
Layer	$n$	$\kappa$	$d$(nm)	$n$	$\kappa$	$d$(nm)	$n$	$\kappa$	$d$(nm)
Inner Ag	$0.0719$ ^a^	$4.1430$ ^a^	41	$0.0719$ ^a^	$4.1430$ ^a^	$33.80$	$0.0719$ ^a^	$4.1430$ ^a^	$41.32$
PMMA	$1.4889$ ^a^	0	230	$1.4889$ ^a^	$0.0094$ ^a^	$241.00$	$1.4889$ ^a^	$0.0094$ ^a^	$254.80$
Outer Ag	0.0719^a^	4.1430^a^	34	$0.0719$ ^a^	$4.1430$ ^a^	$46.60$	$0.0719$ ^a^	$3.2150$ ^a^	$46.17$
DR1/PMMA	$1.5650$ ^b^	0	[0–70]	–	–	–	$1.5650$ ^b^	$0.0120$ ^b^	58.90

*Optimized lossless structure.

^a^This paper.

^b^From^10^.

### Photo-induced change of the refractive index of the sensing layer (SL) and comparison of the sensitivities for MIM and CSPR structures

**Figure 5:**
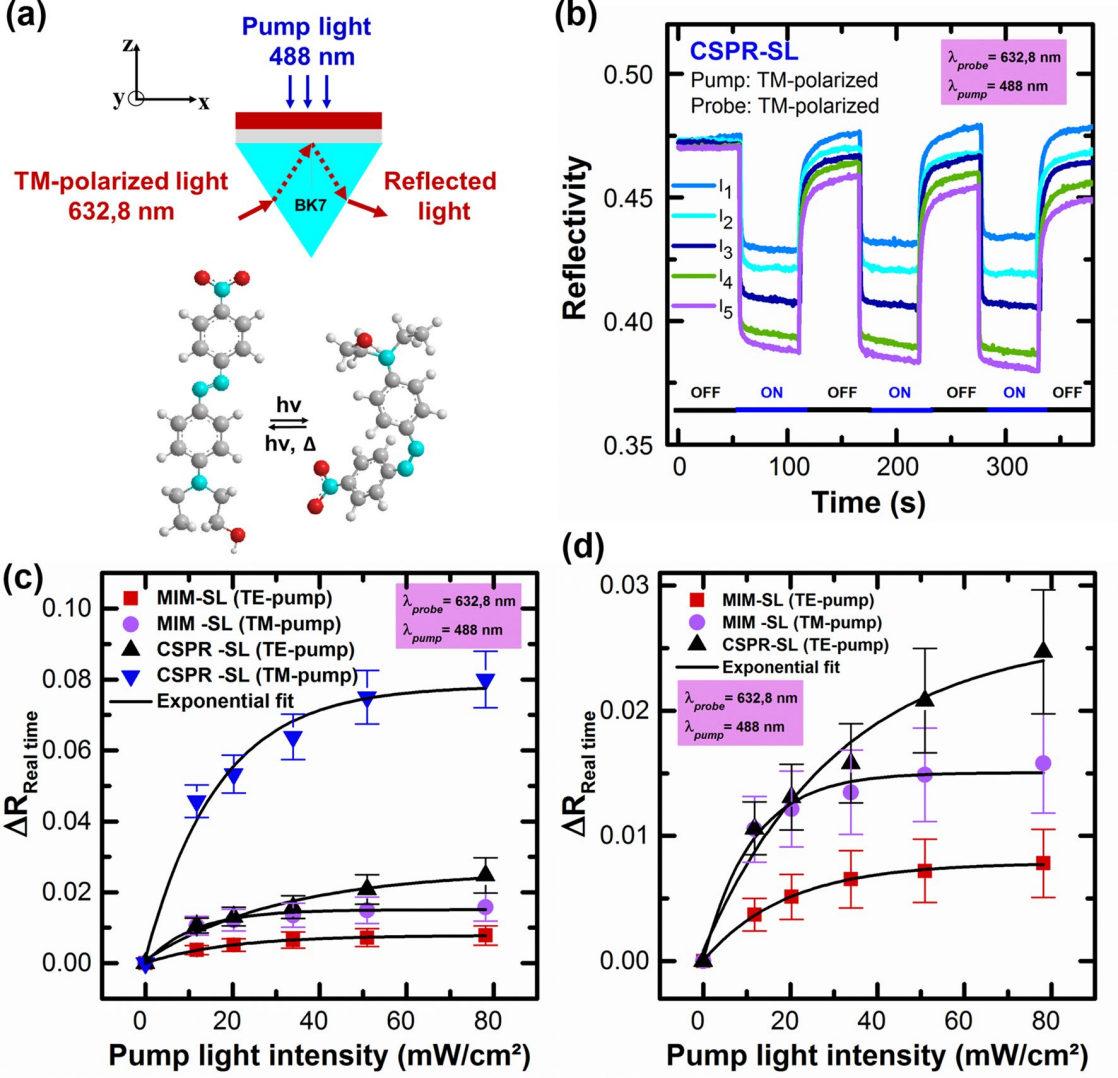
(**a**) Schematics of pump-probe experimental configuration and $trans \leftrightarrow cis$ isomerization of DR1. (**b**) TM mode reflectivity cycling of the CSPR mode by photoisomerization of DR1 in the sensing layer (SL) at five different pump intensities, i.e.${I}_{1}= 11.77 \, {{\text{mW/cm}}^2},$ ${I}_{2} = 20.30 \, {{\text{mW/cm}}^2}$, ${I}_{3} = 33.91\, {{\text{mW/cm}}^2}$, ${I}_{4}= 50.92 \, {{\text{mW/cm}}^2},$ ${I}_{5}= 78.16 \, {{\text{mW/cm}}^2}$. The on–off cycles of the pump light are indicated. (**c**) ∆R measurements of the probe TM mode reflectivity change of MIM and CSPR under TE and TM pump irradiation, and (**d**) is the zoom of (**c**) for small values of ∆R.

We will go on to compare the sensitivities of MIM and CSPR for thin film sensing for a given, experimentally induced ${\Delta n}_{SL}$. First, we study the dynamics of ${\Delta n}_{SL}$, i.e. of photochemical sensing, by real time measurements of reflectivity change under pump light irradiation as indicated in Fig. 5. Pump light induces reversible photoisomerization of DR1 in the SL and changes dynamically the refractive index of that layer, thereby changing the reflectivity. The theoretical base groundwork behind the dynamical change of the refractive index can be found in^55^. The dynamics are recorded by setting the goniometer angle near the resonance mode of the structure, i.e. near the angle which corresponds to the minimum of reflectivity, and recording continuously the change in reflectivity, much like in biomolecular interaction analysis sensing where reflectivity changes are induced by biomolecular interaction which changes the optical thickness of the sample, in our case, the latter is controlled by changing the refractive index of the SL keeping its thickness fixed.

**Figure 6:**
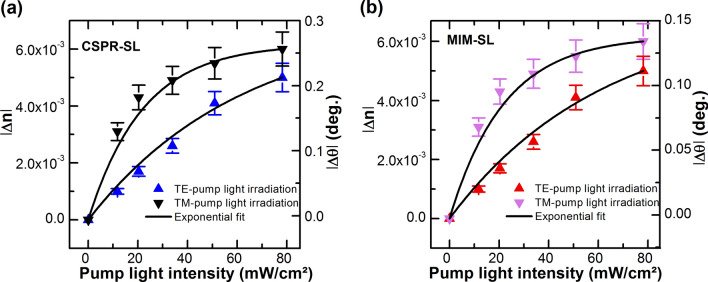
Photoinduced shift of (**a**) CSPR and (**b**) MIM sensors. The right vertical axis represents the shift in the resonance angle and the left axis represents the refractive index change.

In practice, the SL is irradiated with a TM- or TE-polarized pump light with intensities ranging from $11.77 \, {{\text{mW/cm}}^2},$ to $78.16 \, {{\text{mW/cm}}^2},$. During the pumping process, the real time of the photo-induced change of the reflectivity of the probe light at the CSPR and MIM structures, is measured at an angle which is located in the linear part of the resonance mode of the non-irradiated structure, i.e. $58.15^\circ$ and $54.74^\circ$ for CSPR and MIM; respectively, to maximize the response to photo-induced ${\Delta n}_{SL}$. Figure 5b shows reversible cycle changes of the reflectivity of CSPR sample under TM-pump irradiation with five different intensities, i.e. ${I}_{1}= 11.77 \, {{\text{mW/cm}}^2},$, ${I}_{2} = 20.30 \, {{\text{mW/cm}}^2},$, ${I}_{3} = 33.9 \, {{\text{mW/cm}}^2},$, ${I}_{4}= 50.92 \, {{\text{mW/cm}}^2},$ and ${I}_{5}= 78.16 \, {{\text{mW/cm}}^2},$. The moments of turning the irradiation light ON and OFF are indicated in the figure. By increasing the light intensity of the pump beam irradiation, we observed that the response is increasing with the increased $\Delta {n}_{SL}$. During the photoisomerization process, the configuration of DR1 molecules changes from the trans-to-cis (Fig. 5a) and the polarizability of the molecules changes as well; a feature that leads to the changes, i.e. decrease, in the refractive index of DR1/PMMA sensing layer, and as a consequence the mode of the plasmonic structure shifts to smaller resonance angles and the reflectivity decreases (Fig. 5). The reversibility of $\Delta {n}_{SL}$ is due to the reversible, thermally activated, cis–trans isomerization, i.e. from the cis- to the thermally more stable trans-isomer. Similar dynamical behavior is observed for the MIM sensor (not shown), and the changes in reflectivity and $\Delta n$ and $\Delta \theta$ are shown in Fig. 6a,b for both MIM and CSPR structures.

The $\Delta R$ response [calculated by using Eq. (1)] is determined based on real time measurements with and without pump irradiation. By increasing the intensity of the pump beam irradiation ∆R increases owing to decreased $\Delta {n}_{SL}$ and mode shift. TE-polarized pump irradiation leads to similar dynamical behavior but with different efficiencies depending on the pump intensity. Indeed, as well known from a long literature^23,26^, azo dye molecules are polarization sensitive, and they are photo-selected and photo-oriented by polarized light irradiation by undergoing shape change and reorientation. The related refractive index change for parallel (perpendicular) pump-probe polarizations irradiation ${{\Delta n}_{par}(\Delta n}_{per})$ is ${(\Delta n}_{par}\sim 3{\Delta n}_{per}$) for small irradiation intensities, and ${(\Delta n}_{par}\sim {\Delta n}_{per})$ for large irradiation intensities according to the orientational hole burning (OHB) model^25^. The latter states that polarized-pump irradiation, burns a hole into the orientational distribution of the molecules, owing to photoselection^25,28^. The OHB model behavior is clearly seen in Fig. 6 where ${\Delta n}_{par}\sim 3{\Delta n}_{per}$ for low intensities and ${(\Delta n}_{par}\sim {\Delta n}_{per})$ for high intensities of irradiation; a behavior which is observed for both MIM and CSPR sensors. The differing sensitivities of CSPR and MIM sensors can be seen in both Figs. 5c and 6. Indeed, for the same $\Delta n$, which is a property of the SL, not the sensor, we observe larger reflectivity changes ($\Delta R$ in Fig. 5c) and mode shifts ($\Delta \theta$ in Fig. 6) for CSPR versus MIM sensors. It can be seen from both Figs. 5c and 6, that the response of CSPR compared to that of MIM is $\sim 3$ to 5 times larger; respectively. These experimental results confirm our theoretical predictions on both sensors. That is, CSPR is superior to MIM for thin film sensing.

In summary, electromagnetic calculations of the electric field profile together with sensitivity calculations demonstrate that a thin adsorbate layer decreases the sensitivity of MIM compared to CSPR plasmonic structure. The adsorbate modifies the relative contributions of the Ag/Air-SPP and S-SPP modes to the coupled modes of the MIM structure; a feature which reduces the electric field both inside the adsorbate layer and at the interface of this layer with air, thereby decreasing the sensitivity to refractive index changes of the adsorbate layer. Experimental results based on artificial refractive index changes of the adsorbate layer by a photochemical reaction, in both MIM and CSPR structures, which causes mode shifts and reflectivity changes in both CSPR and MIM ATR spectra, allowed for the comparison of the sensitivities for both structures and showed that CSPR gives a higher response to the refractive index changes compared to MIM, and confirmed the theoretical predictions of sensitivity calculations. While in previous studies MIM was shown to be $\sim 7$ times better than CSPR for bulk sensing, this study shows that CSPR $3\sim 5$ times better than MIM for thin film sensing; a feature which is important in sensors design and applications.

## Supplementary Information

Supplementary Information.

## Data Availability

The authors declare that all data supporting the findings of this study are available from the corresponding author upon reasonable request.
