# Supplementary material for: Plasmonic mode coupling and thin film sensing in metal–insulator–metal structures
Source: Sci Rep. 2021 Jul 23;11:15093. doi: 10.1038/s41598-021-94143-2 (PMC8302593; doi:10.1038/s41598-021-94143-2)
Supplement: Supplementary file 1 — Supplementary Information. [file 41598_2021_94143_MOESM1_ESM.docx]

**Supporting Information**

**Plasmonic Mode Coupling and Thin Film Sensing**

**in Metal-Insulator-Metal Structures**

**N. Andam^1,2^, S.Refki^2^, S.Hayashi^2,3^ and Z.Sekkat^1,2,4^***

^1^Department of Chemistry, Faculty of Sciences, University Mohammed V, Rabat, Morocco;

^2^Optics and Photonics Center, Moroccan Foundation for Advanced Science and Innovation and Research, University Mohammed VI Polytechnic, Rabat, Morocco;

^3^Graduate School of Engineering, Kobe University, Kobe 657–8501, Japan

^4^ Department of Applied Physics, Osaka University, 2-1 Yamadaoka, Suita, Osaka 565-0871, Japan.

*Corresponding author: z.sekkat@mascir.ma

**Anticrossing behaviors of MIM coupled modes caused by change in the overlayer thickness**

To clarify the physical origin of changes in the electric field profile of the MIM structure brought by the change in the overlayer thickness (Fig. 2(d) in the main text), we have performed EM calculations for MIM structures with different coupling strengths. We demonstrate here the anticrossing behaviors of the coupled modes and study in detail the changes in the electric field profile caused by the variation of the overlayer thickness.

First, we consider the uncoupled Ag/Air-SPP and MIM S-SPP modes. The resonance position of the uncoupled MIM S-SPP mode is known from the ATR calculation of an MIM structure having a semi-infinite outer Ag layer. In the calculation, we used the same refractive indices and thicknesses for the Ag and PMMA layers (except for the outer Ag layer) as used in the theoretical calculations described in the main text. The ATR spectrum obtained exhibits an ATR dip at an incidence angle of 52.81°. In Fig. S1, this resonance position of the uncoupled MIM S-SPP mode is shown as a horizontal broken line. As discussed in detail in the main text, the ATR dip of the CSPR structure corresponding to the excitation of the Ag/Air-SPP mode shifts to higher angles as the SL thickness, *d*, increases; in Fig. S1, the resonance positions of the Ag/Air-SPP mode are represented by the filled triangles. We see that there is a crossing point of the resonance positions around $d=48 \mathrm{nm}$. As the SL thickness increases, the detuning of the modes (difference in the resonance positions) decreases first, becomes zero around $d=48 nm$ and turns to increase when the thickness increases further.

In the MIM structures considered in the present study, the outer Ag layer has a finite thickness and consequently, the Ag/Air-SPP and MIM S-SPP modes couple through the evanescent tails inside the outer Ag layer. The coupling of the modes results in hybridized modes constructed by in-phase (symmetric) and out-of-phase (antisymmetric) superpositions of the respective modes. The coupling strength is controlled by the thickness of the outer Ag layer, and the thinner the layer, the stronger the coupling. We performed EM calculations for two different MIM structures, one with a 70 nm-thick outer Ag layer working under a weak coupling strength (referred to as W-MIM structure) and other with a 40 nm-thick outer Ag layer working under a strong coupling strength (referred to as S-MIM structure). Except for the thickness of the outer Ag layer, we used the same structural parameters as those in the theoretical calculations presented in the main text. In all the calculated ATR spectra, two ATR dips corresponding to the excitation of the coupled modes appeared and shifted to higher angles as the SL thickness increases. In figure. S1, the positions of the low- and high-angle modes are represented by the filled circles (W-MIM) and squares (S-MIM), respectively. For the W-MIM structure, when the SL is thin ( $d<\sim30 \mathrm{nm}$), the low-angle (high-angle) mode is located close to the uncoupled Ag/Air-SPP (MIM S-SPP) mode, but around the crossing point at $d=48 \mathrm{nm}$ the modes are pushed away and deviate from the uncoupled modes. For thick enough SL ($d>\sim65 \mathrm{nm}$), the low-angle mode approaches the uncoupled MIM S-SPP mode, while the high-angle mode approaches the uncoupled Ag-Air SPP mode. The behavior of the modes seen here is a typical anticrossing behavior caused by the changes in the detuning of the uncoupled modes^1,2^. For the S-MIM structure, since the coupling is stronger, the splitting between the low- and high-angle modes is larger, and the resonance positions of the coupled modes are rather far from the uncoupled modes.





**Fig. S1** Resonance positions of uncoupled MIM S-SPP and Ag/Air-SPP modes, and anticrossing behavior of coupled modes in weak coupling MIM (W-MIM) and strong coupling MIM (S-MIM) structures.

We have calculated the electric field profiles generated in W- and S-MIM structures with SL of $d=30, 45$and 60 nm upon excitation of the coupled modes (points Nos. 1 - 6 and Nos. 1’- 6’ in Fig. S1). In Fig.S2, electric field profiles in the W-MIM structure corresponding to points Nos. 1 - 6 are presented, and in Fig. S3, those in S-MIM structure corresponding to Nos. 1’- 6’ are presented. In Fig. S2, we see that the profile No.1 (No. 2) still conserves the characteristics of the Ag/Air SPP (MIM S-SPP) mode, since the point No. 1 (No. 2) in Fig. S1 is still close to the uncoupled Ag/Air SPP (MIM S-SPP) mode and the effect of coupling on the modes is still weak.


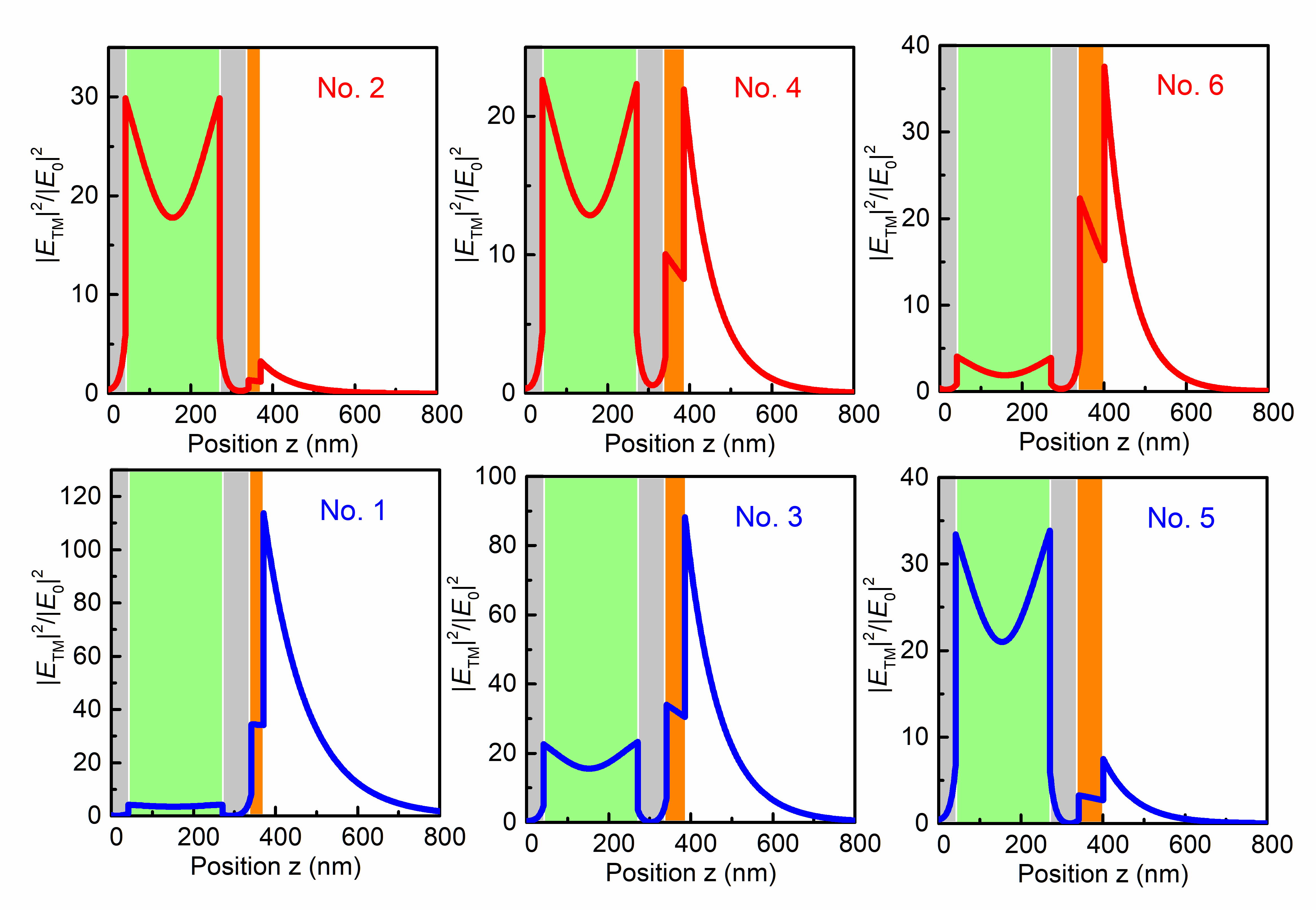


**Fig. S2** Electric field profiles obtained for W-MIM structure corresponding to points Nos. 1 - 6 in Fig. S1.

However, around the crossing point, the effect of coupling is stronger and the profiles Nos. 3 and 4 clearly exhibit hybrid nature of the modes, where both uncoupled modes contribute significantly to the profiles; for the profile No. 4, we see almost equal contributions of the modes. In the profiles No. 5 and No. 6, we see strong contributions of the MIM S-SPP and Ag/Air-SPP mode, respectively, in accordance with the fact that the points No. 5 and No. 6 are close to the uncoupled MIM S-SPP and Ag/Air-SPP mode, respectively.


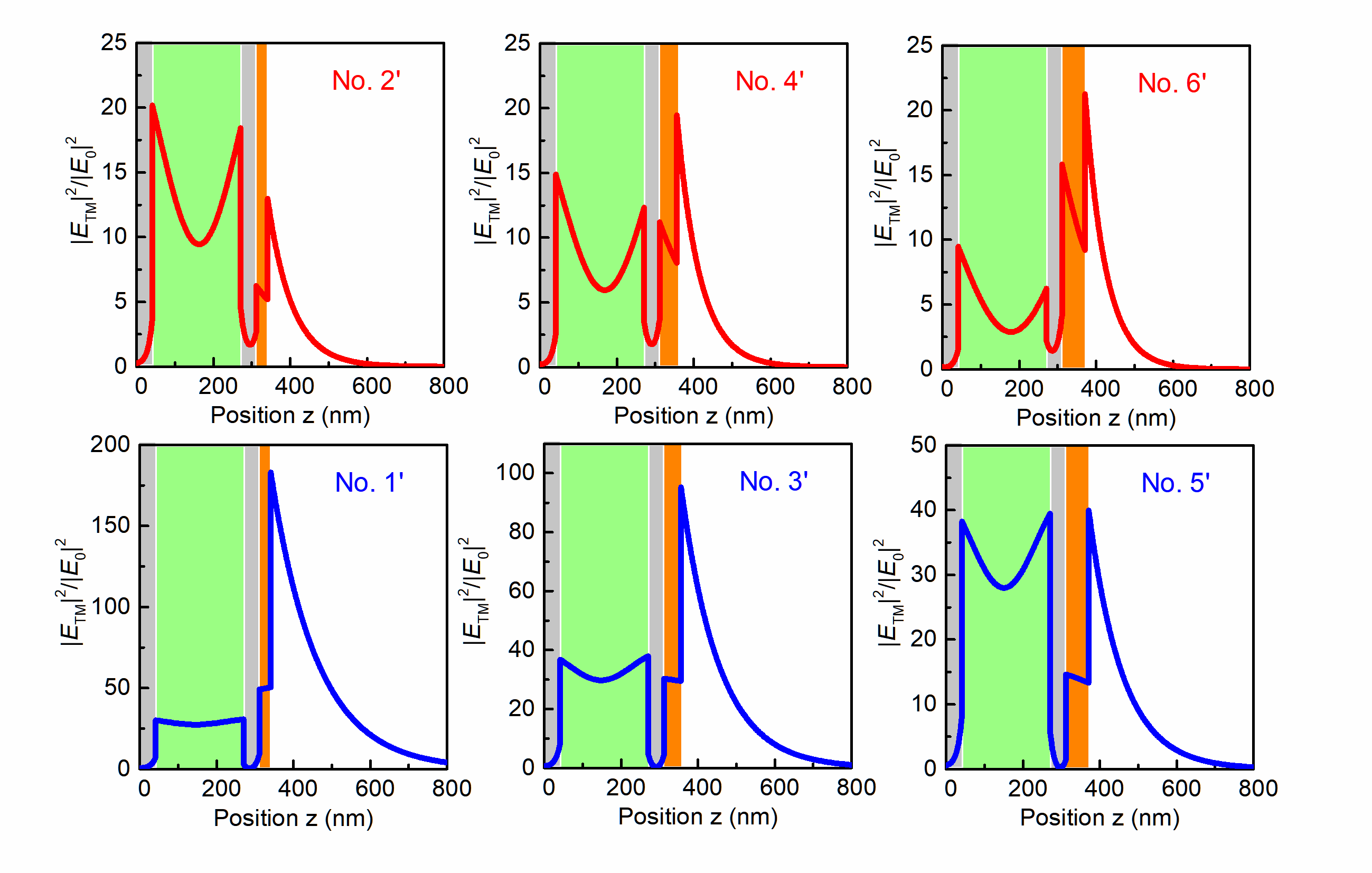


**Fig. S3** Electric field profiles obtained for S-MIM structure corresponding to points Nos. 1’ – 6’ in Fig. S1.

For the S-MIM structure in which the coupling is stronger, the profiles Nos. 1’ - 6’ presented in Fig. S3 demonstrate again the hybrid nature of the modes and the change in the relative contributions of the MIM S-SPP and Ag/Air-SPP modes caused by the change in the detuning. It should be noted that in the profiles Nos. 1’, 3’ and 5’ corresponding to the low-angle modes in Fig. S1, the electric field amplitude goes down to zero around the center of the outer Ag layer; this is caused by the out-of-phase superposition of the evanescent tails inside the layer (antisymmetric mode). In contrast, in the profiles Nos. 2’, 4’ and 6’, the electric field amplitude inside the outer Ag layer does not go down to zero, because the evanescent tails are superposed in-phase. The electric field profiles shown in Figs. S2 and S3 demonstrate that relative contributions of the MIM S-SPP and Ag/Air-SPP modes to the coupled modes in MIM structures depend strongly on the coupling strength and the detuning of the uncoupled modes.

The thickness of the outer Ag layer used in the theoretical calculation of the MIM structure in the main text is $d=34$ nm, slightly smaller than that of the above S-MIM structure. Although the coupling is stronger than the S-MIM structure, the hybrid nature of the coupled mode (lower-angle mode) seen in Fig. 2(d) is essentially the same as that seen in profiles Nos. 1’, 3’ and 5’ in Fig. S3. We can finally conclude that the drastic changes of the electric field profile seen in Fig. 2(d) reflect the strong dependence of the MIM coupled mode on the detuning of the uncoupled modes.

**References**

1. Novotny, L. Strong coupling, energy splitting, and level crossings: A classical perspective. *Am. J. Phys.* **78**, 1199–1202 (2010).

2. Refki, S., Hayashi, S., Rahmouni, A., Nesterenko, D. V. & Sekkat, Z. Anticrossing behavior of surface plasmon polariton dispersions in metal-insulator-metal structures. *Plasmonics* **11**, 433–440 (2016).
